# A 3‐week multimodal intervention involving high‐intensity interval training in female cancer survivors: a randomized controlled trial

**DOI:** 10.14814/phy2.12693

**Published:** 2016-02-11

**Authors:** Joachim Schmitt, Nathalie Lindner, Monika Reuss‐Borst, Hans‐Christer Holmberg, Billy Sperlich

**Affiliations:** ^1^ Rehaklinik am Kurpark, RehaZentren Baden Württemberg gGmbHBad KissingenGermany; ^2^ Integrative and Experimental Training ScienceDepartment of Sport ScienceJulius‐Maximilians‐Universität WürzburgWürzburgGermany; ^3^ Swedish Winter Sports Research CentreDepartment of Health SciencesMid Sweden UniversityÖstersundSweden; ^4^ School of KinesiologyUniversity of British ColumbiaVancouverBCCanada

**Keywords:** Cancer survivors, cardiorespiratory fitness, energy expenditure, exercise, rehabilitation, sense wear

## Abstract

To compare the effects of a 3‐week multimodal rehabilitation involving supervised high‐intensity interval training (HIIT) on female breast cancer survivors with respect to key variables of aerobic fitness, body composition, energy expenditure, cancer‐related fatigue, and quality of life to those of a standard multimodal rehabilitation program. A randomized controlled trial design was administered. Twenty‐eight women, who had been treated for cancer were randomly assigned to either a group performing exercise of low‐to‐moderate intensity (LMIE;* n* = 14) or a group performing high‐intensity interval training (HIIT;* n* = 14) as part of a 3‐week multimodal rehabilitation program. No adverse events related to the exercise were reported. Work economy improved following both HIIT and LMIE, with improved peak oxygen uptake following LMIE. HIIT reduced mean total body fat mass with no change in body mass, muscle or fat‐free mass (best *P* < 0.06). LMIE increased muscle and total fat‐free body mass. Total energy expenditure (*P* = 0.45) did not change between the groups, whereas both improved quality of life to a similar high extent and lessened cancer‐related fatigue. This randomized controlled study demonstrates that HIIT can be performed by female cancer survivors without adverse health effects. Here, HIIT and LMIE both improved work economy, quality of life and cancer‐related fatigue, body composition or energy expenditure. Since the outcomes were similar, but HIIT takes less time, this may be a time‐efficient strategy for improving certain aspects of the health of female cancer survivors.

## Introduction

Cancer survivors often suffer seriously from impaired health‐related quality of life, including reduced cardiorespiratory and metabolic fitness and weight gain associated with cancer‐related fatigue, as well as lower psychophysical health and higher premature mortality (Courneya and Friedenreich 2001). Recent meta‐analyses indicate that energy expenditure improves the body composition, psychophysical health, and quality of life in such patients (Speck et al. 2010; Fong et al. 2012). In this context, another meta‐analysis revealed that low‐intensity walking potentially ameliorates cancer‐related fatigue (Kangas et al. 2008).

Attempts to improve cardiovascular and metabolic health in individuals with a variety of diseases have begun to replace low‐intensity exercise with repeated short‐to‐long bouts of rather high‐intensity exercise interspersed with recovery periods (referred to as high‐intensity interval training [HIIT]) (Kessler et al. 2012; Elliott et al. 2014; Little and Francois 2014; Gielen et al. 2015), with no adverse consequences mentioned so far. For example, when used as an adjunct to chemotherapy, resistance and aerobic exercise of moderate‐to‐high intensity reduced fatigue and improved vitality, aerobic capacity, muscular strength, physical and functional activity, emotional well‐being, and quality of life (Adamsen et al. 2009; van Waart et al. 2015).

A 12‐week random‐controlled intervention involving high‐intensity exercise by cancer survivors proved to be safe and effective in reducing general and physical fatigue (Kampshoff et al. 2015) and 4 weeks of high‐intensity exercise improved both the cardiorespiratory fitness and body composition of patients who had survived colorectal cancer (Devin et al. 2015). Another random‐controlled study showed that 8 weeks of individualized, high‐intensity aerobic interval training improves circulatory, respiratory, and muscular function at peak exercise, with less dyspnea and lower fatigue, in patients with non‐small cell lung cancer (Hwang et al. 2012).

Moreover, a 12‐month exercise‐based rehabilitation program involving once weekly high‐intensity group‐based exercise for patients with different types of cancer improved self‐reported energy expenditure, cardiorespiratory fitness (VO_2peak_), strength, and mental health (Midtgaard et al. 2013). Finally, a single‐blind randomized controlled intervention of high‐intensity endurance and strength training (60 min, three times a week, 20 weeks) for patients with lung cancer, starting 5–7 weeks after surgery in conjunction with standard postoperative care, was well tolerated and resulted in clinically significant improvements in VO_2peak_, muscular strength, total muscle mass, functional fitness, and quality of life (Edvardsen et al. 2015).

Furthermore, a recent pilot study concluded that 5 weeks of high‐intensity functional training was both well received and beneficial for emotional functioning, body composition, and functional movement (Heinrich et al. 2015). In this context, exercise of higher intensity was found to help sustain the motivation of cancer survivors to exercise (Martin et al. 2015).

Because these previous studies varied in design with respect to the type of exercise, whether it was performed together with or after chemotherapy, the type of cancer and gender, no general conclusions concerning the benefits of HIIT for cancer patients can be drawn. Therefore, the psychophysiological effects of a 3‐week multimodal rehabilitation program including supervised HIIT on key variables of aerobic fitness, body composition, energy expenditure, quality of life, and cancer‐related fatigue in female cancer survivors was compared to those of a standard multimodal program of rehabilitation. Since HIIT has proven to be a time‐efficient intervention in various diseased population, we hypothesized greater health‐related benefits with HIIT in female cancer survivors when compared to a 3‐week multimodal rehabilitation program.

## Methods

A single‐center, two‐arm randomized controlled design was employed, with each participant being randomly assigned by the study coordinator according to a simple computer‐generated randomization schedule (http://www.randomization.com/) before the first testing to either the group performing high‐intensity interval training (HIIT) or the control group undergoing traditional exercise of low‐to‐moderate intensity (LMIE).

From *n* = 40 volunteers, 26 female cancer survivors were recruited in 2014 from their health insurance cancer‐related medical program and implemented into the study according to the inclusion and exclusion criteria which were defined a priori (Table 1). *N* = 14 females did not meet the eligibility criteria.

**Table 1:** The inclusion and exclusion criteria of this study

Inclusion criteria	Exclusion criteria
‐ Diagnoses of cancer (ICD‐10 C00–D48) ‐ Female ‐ Completion of chemotherapy or radiation ‐ Body mass index 17–35	‐ Age <18 years, >70 years ‐ Bone metastases ‐ symptomatic cardiac disease ‐ contraindication to maximal exercise testing and/or exercise training

The anthropometric and medical characteristics of the 28 female cancer survivors are presented in Table 2.

**Table 2:** The anthropometric and medical characteristics (number or mean ± SD) as well as the energy expenditure at different intensities of the participants

	Group	
	HIIT (*n* = 13)	LMIE (*n* = 13)
Age in years	53 ± 8	54 ± 9
Body mass index [kg/m^2^]	27.0 ± 5.3	26.2 ± 4.3
Months after initial diagnosis	14 ± 20	25 ± 70
Recurrent tumor	1 (8%)	2 (15%)
Type of tumor		
Breast	11 (85%)	10 (77%)
Ovarian	1 (8%)	0
Colon	0	1 (8%)
Vaginal	0	1 (8%)
Non‐invasive urothelial carcinoma	1 (8%)	0
Non‐Hodgkin′s lymphoma	0	1 (8%)
Metastases	2 (15%)	1 (8%)
Treatment		
Surgical	13 (100%)	13 (100%)
Chemotherapy	7 (54%)	9 (69%)
Months since chemotherapy	15 ± 8	11 ± 37
Radiation	9 (69%)	9 (69%)
Months since last radiation	13 ± 8	5 ± 14
Antihormonal therapy	9 (69%)	7 (54%)
Still undergoing		
Antihormonal therapy	7 (54%)	5 (39%)
Activity and energy expenditure during intervention		
Step counts	12.520 ± 2.746	12.400 ± 2.630
Total energy expenditure [kcal]	2.362 ± 294	2.282 ± 237
Energy expenditure >3 MET [kcal]	470 ± 205	413 ± 168
Duration of activity >3 MET [min]	108 ± 49	98 ± 40
Duration of activity 0–3 MET min]	223 ± 110	225 ± 71
Duration of activity 3–6 MET [min]	106 ± 46	97 ± 39
Duration of activity 6–9 MET [min]	2 ± 4	1 ± 1
Duration of activity >9 MET [min]	1 ± 2	0 ± 0

All were informed about the design of the study, with information concerning possible risks and benefits, before providing written informed consent to participate. All procedures were performed in accordance with the Declaration of Helsinki and the guidelines of the Ethical Committee of the Department of Sport Science in Würzburg, Germany.

German insurance guidelines normally specify 3 weeks of multimodal rehabilitation for cancer survivors, including medical and therapeutic treatment, as well as mandatory educational sessions. During this period, the patients in both of our groups took part in numerous sessions concerning nutrition, psychological and general health, occupational therapy, social counseling, and relaxation in accordance with these national guidelines. Only the program of exercise differed.

### Intervention

As performed by previous health‐related studies (Kessler et al. 2012; Elliott et al. 2014; Little and Francois 2014; Gielen et al. 2015), our patients completed a total of eight sessions of HIIT (three sessions per week, separated by at least 24 h) on a paved up‐hill road. All sessions were designed as an outdoor group session with individual prescription of intensity. After warming up for 5 min at 70% of peak heart rate (HR_peak_) (determined by an initial incremental treadmill test), each performed eight 1‐min sessions at >95% HR_peak_ of intense walking interspersed by 2‐min intervals of slow walking as recovery. Heart rate was monitored telemetrically (Polar, FS1c, Polar Oy, Kempele, Finland).

Based on international recommendations (Schmitz et al. 2010), the participants in the control group performed six 75‐min sessions of moderate intensity in a group including 60 min of walking outdoors and 15 min indoor cycling at 60% HR_peak_ (as monitored by a therapist) over a three‐week period.

### Pre‐ and postdiagnostic

All baseline values were obtained 1 day before and all post data 2 days after the 3 weeks of multimodal rehabilitation by the same medical staff.

Oxygen uptake, heart rate, the respiratory exchange ratio, and blood level of lactate were assessed before and during an incremental treadmill (Therapie, Sprintex, Germany) protocol in accordance to previous recommendations (Bruce et al. 1973) starting at 2.7 km/h and no incline, with increases in one or both of these every 3 min.

Oxygen uptake was measured continuously throughout the treadmill testing with an open circuit breath‐by‐breath gas analyzer (Aeromen, Aerolution, Fürth, Germany) employing standard dynamic algorithms to correct for the time delay between the gas consumption and signal. All respiratory data were averaged every 30 sec. Submaximal oxygen uptake at 2.7 km/h was considered to represent running economy, as described elsewhere (Barnes and Kilding 2015). The highest value for oxygen uptake during the last 30 s of the test was defined as VO_2peak_.

Body composition (lean and fat mass) was determined by dual‐energy X‐ray absorptiometry (DXA; HOLOGIC^®^, Explorer S/N 90425, software version 12.4). Body mass was determined with personal scales (KERN MPE250K100PM, Germany).

The Bodymedia^®^ Sensewear^™^ Armband provided an objective measurement of energy expenditure both during the intervention and free time, beginning on the 8th and ending on the 15th day of the program. This armband integrates a biaxial accelerometer with sensors that monitor skin temperature, near‐body temperature, heat flux, and galvanic skin response, and has been validated previously as reliably estimating the total energy expenditure of women moving freely (Farooqi et al. 2013).

Before and after the intervention, the Multidimensional Fatigue Inventory (MFI‐20) was used to assess general, physical, and mental fatigue, as well as activity and motivation (Smets et al. 1995). The total score for each subscale ranges from 4 to 20, with higher scores indicating more pronounced fatigue (Smets et al. 1995; Lin et al. 2009).

Before and after the intervention, all of the women were asked to complete the EORTC core instrument (QLQ‐C 30) on their own (Aaronson et al. 1993) 1 day before and 1 day after the last treatment. The EORTC QLQ‐C 30 (version 3.0) consists of five functional scales (physical, role, emotional, cognitive, and social) and nine subscales concerning symptoms (fatigue, nausea and vomiting, pain, dyspnea, insomnia, loss of appetite, constipation, diarrhea, and financial difficulties) due to poor health and/or the disease; for further details, see (Aaronson et al. 1993).)

For all data, mean values and standard deviations (SD) were calculated in the conventional manner. Normal distribution was confirmed with the Kolmogorov–Smirnov test and homogeneity of variance verified by Levine's test, so that no further transformation was necessary. The effect size Cohen's *d* (Cohen 1988) between pre‐ and posttesting, was calculated for all variables with the thresholds for small, moderate, and large effects being 0.20, 0.50, and 0.80, respectively. Repeated‐measures ANOVA was used to compare each variable at these two time points. When a global difference over time was observed, Bonferroni post hoc analysis was applied to identify when the changes occurred. An alpha of *P *<* *0.05 was considered statistically significant and indicated as *. All statistical tests were carried out in the SPSS Statistics (version 19, IBM, Ehningen, Germany) software.

## Results

A total of 93% of the participants in both groups completed all of the training sessions, for a total average duration of endurance exercise of 239 ± 17 and 413 ± 32 min for HIIT and LMIE, respectively. None of the participants reported negative side effects related to the intervention; therefore, none of the session had to be terminated.

The data of all variables as well the magnitude of outcomes are summarized in Table 3.

**Table 3:** All mean (±SD) data and statistical analysis of the participants

	High‐intensity interval group (*n* = 13)		Low‐to‐moderate intensity group (*n* = 13)		*P*‐value (effects size) pre to post		*P*‐value (effects size) between group effect
	pre	post	pre	post	HIIT	LMIE	
Peak oxygen uptake [mL/kg/min]	27.1 ± 7.9	27.0 ± 7.3	23.8 ± 5.0	26.3 ± 5.6	0.42 (−0.02)	<0.00*(0.46)	0.01*(1.27)
Oxygen uptake at 2.7 km/h [mL kg/min]	14.6 ± 3.7	13.1 ± 2.6	13.6 ± 1.9	13.0 ± 1.8	0.02*(−0.23)	0.18 (−0.16)	0.34 (−0.38)
Body mass [kg]	73.5 ± 14.3	73.1 ± 14.2	70.8 ± 13.6	70.8 ± 13.6	0.06 (−0.03)	0.48 (0.00)	0.22 (−0.18)
Fat mass [kg]	27.4 ± 11.1	26.9 ± 10.5	26.9 ± 10.0	25.5 ± 11.6	0.02*(−0.05)	0.06 (−0.13)	0.31 (0.15)
Muscle mass [kg]	44.1 ± 4.9	44.1 ± 5.1	41.7 ± 4.9	42.2 ± 4.8	0.40 (0.01)	0.04*(0.10)	0.24 (−0.17)
Fat‐free mass [kg]	46.1 ± 5.1	46.2 ± 5.3	43.9 ± 5.0	44.4 ± 5.0	0.38 (0.01)	0.03*(0.10)	0.24 (−0.17)
General fatigue [a.u.]	11.6 ± 2.9	10.3 ± 2.9	14.5 ± 3.6	11.3 ± 3.5	0.02*(−0.45)	<0.00*(−0.90)	0.04*(0.30)
Physical fatigue [a.u.]	10.8 ± 3.5	9.2 ± 2.8	14.1 ± 3.1	12.5 ± 4.5	0.01*(−0.52)	0.06 (0.44)	0.95 (0.01)
Mental fatigue [a.u.]	12.2 ± 4.6	10.1 ± 4.0	12.7 ± 3.9	11.4 ± 3.2	0.01*(−0.46)	0.07 (−0.37)	0.50 (−0.09
Reduced activity [a.u.]	10.3 ± 3.3	8.6 ± 2.6	12.1 ± 2.6	10.2 ± 2.2	0.01*(−0.55)	0.01*(−0.80)	0.80 (0.04)
Reduced motivation [a.u.]	12.0 ± 2.8	9.4 ± 3.2	11.2 ± 2.1	9.5 ± 2.6	<0.00*(−0.85)	0.01*(‐0.72)	0.34 (−0.13)
Quality of life [a.u.]	63.5 ± 14.1	73.0 ± 11.2	40.9 ± 11.0	58.3 ± 18.6	<0.00*(0.79)	<0.00*(1.14)	0.09 (−0.24)
Physical functioning [a.u.]	83.1 ± 12.5	87.2 ± 11.0	68.8 ± 16.9	81.5 ± 11.3	0.04*(0.35)	<0.00*(0.89)	0.07 (−0.28)
Social functioning [a.u.]	69.1 ± 27.0	66.7 ± 28.9	49.9 ± 28.1	67.5 ± 28.4	0.33 (−0.08)	0.01*(0.64)	0.02*(−0.34)
Emotional functioning [a.u.]	60.9 ± 26.0	71.9 ± 18.7	39.7 ± 24.6	71.2 ± 25.9	0.03*(0.46)	<0.00*(1.25)	0.03*(−0.32)
Cognitive functioning [a.u.]	60.3 ± 25.8	69.2 ± 29.5	60.3 ± 28.4	73.1 ± 24.0	0.07 (0.31)	0.04*(0.48)	0.66 (−0.06)
Role functioning [a.u.]	65.5 ± 25.8	88.5 ± 17.1	41.0 ± 28.0	71.9 ± 22.0	0.01*(1.04)	<0.00*(1.23)	0.45 (−0.10)
Fatigue [a.u.]	39.2 ± 20.3	27.1 ± 13.9	68.5 ± 19.7	38.3 ± 21.7	0.02*(−0.67)	<0.00*(−1.46)	0.02*(0.35)
Nausea [a.u.]	5.1 ± 12.4	0.00 ± 0.00	12.9 ± 18.2	5.1 ± 14.3	0.09 8 (−0.53)	0.10 (−0.47)	0.58 (0.08)
Pain [a.u.]	20.5 ± 23.7	19.2 ± 20.2	55.1 ± 30.6	32.0 ± 18.6	0.42 (−0.07)	<0.00*(−0.91)	0.02*(0.34)
Dyspnea [a.u.]	25.7 ± 31.0	20.3 ± 16.7	53.9 ± 37.4	33.2 ± 23.7	0.21 (−0.22)	0.03*(−0.66)	0.20 (0.18)
Insomnia [a.u.]	48.8 ± 29.4	38.5 ± 26.9	61.5 ± 26.9	43.5 ± 25.2	0.02*(−0.35)	0.01*(−0.69)	0.32 (0.14)
Loss of appetite [a.u.]	7.7 ± 20.0	2.5 ± 9.2	20.5 ± 25.6	7.6 ± 14.5	0.20 (−0.32)	0.04*(−0.62)	0.43 (0.11)
Constipation [a.u.]	7.6 ± 14.5	7.7 ± 20.0	12.7 ± 16.7	7.7 ± 20.0	0.35 (0.00)	0.36 (−0.27)	0.50 (0.10)
Diarrhea [a.u.]	5.1 ± 12.4	5.1 ± 12.4	33.3 ± 33.4	15.3 ± 29.2	0.50 (0.00)	0.05 (−0.57)	0.16 (0.20)
Financial concerns [a.u.]	28.2 ± 42.7	25.6 ± 33.8	38.5 ± 35.7	40.5 ± 38.1	0.42 (−0.06)	0.41 (0.05)	0.69 (−0.06)

*Significant for *P* < 0.05.

The peak oxygen uptake was improved following LMIE (*P* < 0.01). At the same time, work economy (as reflected by oxygen uptake at 2.7 km/h) was improved to a greater extent by HIIT than LMIE (*P* = 0.02).

The women who performed HIIT exhibited no change in total body mass, fat‐free mass, muscle mass (best *P* < 0.06), but a reduction in mean total body fat mass by 531 ± 692 g (*P* = 0.02). The women who carried out LMIE increased both their muscle (*P* = 0.04) and total fat‐free body mass (*P* = 0.03). There were no significant differences between the intervention groups with respect to body composition.

The participants in both groups wore the SenseWear^®^ armband for 96% of the time during the 7‐day investigation (day 7 to day 13 of the intervention). The mean data of both interventions are summarized in Table 2. With regard to total energy expenditure (*P* = 0.45), total active energy expenditure (*P* = 0.44), duration of activities in different MET intervals of exercise (0–3 METs, *P* = 0.96; 3–6 MET, *P* = 0.61; 6–9 MET, *P* = 0.20; >9 MET, *P* = 0.76), total number of steps (*P* = 0.91), and average metabolic equivalent (MET; *P* = 0.96), the groups were similar. Nor did the daily active energy expenditure (470 ± 205 kcal for HIIT vs. 413 ± 168 kcal for LMIE; *P* = 0.440), total daily energy expenditure (2362 ± 294 vs. 2282 ± 237 kcal/day; *P* = 0.45), or daily number of steps (12,520 vs. 12,400; *P* = 0.91) differ.

The EORTC QLQ‐C30 questionnaire revealed similar significant improvements in quality of life in both groups (*P* < 0.00), with a higher effect size in LMIE (*d* = 1.14) than HIIT (*d* = 0.79). With LMIE, all functional scales showed improvement (*P* < 0.04; *d* > 0.48), while after HIIT physical, emotional, and role function were better (all *P* < 0.04). LMIE, but not HIIT augmented social functioning (LMIE**:** *P* = 0.01, HIIT *P* = 0.33). None of the functional scales demonstrated any significant group difference.

HIIT lead to significant relief of symptoms related to fatigue (*P* = 0.02) and insomnia (*P* = 0.02), while LMIE significantly lowered expression of fatigue (*P* < 0.00), pain (*P* < 0.00), dyspnea (*P* = 0.03), insomnia (*P* = 0.01), and loss of appetite (*P* = 0.04). Of the two, the influence of LMIE on the expression of fatigue (*P* = 0.02) and the sensation of pain (*P* = 0.02) was more beneficial. All other symptoms, including nausea, constipation, diarrhea, and financial concerns, were unaltered by either intervention.

Both HIIT (−1.31 ± 2.02 points; *P* = 0.02) and LMIE (−3.23 ± 2.52 points, *P* < 0.00) improved symptoms of general fatigue. All fatigue‐related scales were significantly better after HIIT (*P* = 0.02), that is, reduced motivation (*P* < 0.00), physical fatigue (*P* = 0.01, reduced activity (*P* = 0.01), general fatigue (*P* = 0.02), and mental fatigue (*P* = 0.01). LMIE improved general fatigue (*P* < 0.00), motivation (*P* = 0.01), and reduced activity (*P* = 0.01). General fatigue was higher following LMIE than HIIT (*P* = 0.04), while the scores for all other fatigue‐related scales were similar (best *P* = 0.34).

## Discussion

The major findings of this randomized controlled study were as follows: (1) work economy was improved after LMIE and HIIT with improved VO_2peak_ after LMIE; (2) neither HIIT nor LMIE altered body composition or the amount of total energy expenditure; (3) both enhanced quality of life to a similar degree, with high effect sizes; (4) both interventions lessened cancer‐related fatigue; and (5) none of the female cancer survivors reported negative side effects of HIIT or LMIE.

Altogether, 93% of all training sessions in this study were completed by the participants in both of our groups (due to scheduling, not the intensity of the intervention) and no HIIT‐related adverse effects were reported. Therefore, this investigation indicates that 3 weeks of HIIT may safely be performed by female breast cancer survivors.

VO_2peak_ is one of the most important determinants of aerobic fitness and numerous researchers have documented the differences in this parameter between individuals of different age, gender, and levels of performance. VO_2peak_ sets the upper limit for ATP production via oxidative phosphorylation and is closely linked to maximal cardiac output and is therefore frequently employed as an indicator of overall cardiorespiratory fitness (Bassett and Howley 2000). Available evidence suggests that HIIT may improve VO_2peak_ in cancer patients. For example, 24 sessions of HIIT (2–5 min at 80% VO_2peak_, with active recovery at 60% VO_2peak_) enhanced VO_2peak_ by 5.3% (Hwang et al. 2012) and 9 h of high‐intensity cardiovascular and resistance training, relaxation, body awareness training, and massage elevated VO_2peak_ by 10.7% in such individuals (Adamsen et al. 2009).

Here, LMIE but not HIIT altered VO_2_peak and we can only speculate as to why such improvement is influenced by genetic factors (Bouchard et al. 1986), and moreover, the extent of training may not have been sufficient to stimulate such adaptation or the females terminated testing before exhaustion. However, submaximal oxygen uptake at 2.7 km/h was reduced following HIIT and LMIE, with more pronounced effects in the former case. Work economy is commonly defined as the steady state uptake of oxygen (in mL/min/kg) at a standard velocity or as the energetic cost of running (mL/min/m) and reduced submaximal VO_2_ is an indicator of improved work economy (Barnes and Kilding 2015). In addition, the blood level of lactate and heart rate at 2.7 km/h were also reduced following both HIIT and LMIE here, confirming better cardiorespiratory and metabolic economy. Thus, we conclude that our 6–8 sessions of HIIT or LMIE improved running economy.

To the best of our knowledge, this is the first examination of the influence of HIIT on the body composition of female cancer survivors. Neither HIIT nor LMIE altered this composition in a manner with practical relevance. Recently, following HIIT women were shown to have 8% less body fat mass and a 3.5% lower waist circumference (Hazell et al. 2014), but these participants were healthy and trained for a longer period of time with more frequent sessions (6 weeks with 4–6 maximal sprints three times each week) (Hazell et al. 2014). As recently described, postdiagnostic weight gain is common and complex, being influenced by age, ethnicity, weight, smoking status, time elapsed since diagnosis, and endocrine‐modulating therapy (Sedjo et al. 2014). Nevertheless, our present findings indicate that 3 weeks of multimodal rehabilitation with either HIIT or LMIE may not be sufficient to improve the body composition of female cancer survivors.

The main difference between HIIT and LMIE concerns the duration and intensity of the intervention. Although past comparisons of HIIT with an equal amount of low‐intensity exercise found differences in total energy expenditure (Adachi et al. 1996; Jensen et al. 1996), here we observed no such differences, together with similar total energy expenditures per week with HIIT and LMIE. Since previous studies have reported that the SenseWear Armband underestimate energy expenditure in connection with exercise of very high intensities (Drenowatz et al. 2013), we cannot be absolutely certain that energy expenditure with our HIIT and LMIE was similar.

Employing a study design very similar to ours, Adamsen et al. (2009) found that a 6‐week multimodal HIIT intervention for tumor patients undergoing adjuvant chemotherapy did not improve quality of life (Adamsen et al. 2009). Similarly, another investigation reported a positive trend, but no significant increase in the quality of life of cancer patients. In contrast, others (Buffart et al. 2013; Midtgaard et al. 2013) have observed improved quality of life following HIIT interventions.

One possible explanation for such discrepant findings may be the intervention periods utilized, which ranged from 18 weeks to as long as 1 year (Buffart et al. 2013; Midtgaard et al. 2013). Nonetheless, this study demonstrates that as little as 3 weeks of HIIT and LMIE may improve quality of life significantly. However, this effect cannot be attributed solely to the endurance training, since the women lived for these 3 weeks in a “special” environment, receiving mental health care, as well as nutritional and social counseling. All forms of multimodal rehabilitation may help to improve the psychosocial well‐being and quality of life of cancer patients and alleviate symptoms, although active intervention has been shown to be of considerable importance for elevated quality of life in breast cancer survivors (Battaglini et al. 2014).

Previous research has documented reduced cancer‐related fatigue following HIIT (Adamsen et al. 2009; Ferlay et al. 2013; Battaglini et al. 2014) and the present multimodal HIIT rehabilitation produced small‐to‐moderate effects on all scales of the MFI‐20 and EORTC QLQ‐C30 questionnaires. The patients performing LMIE also reported significant benefits with respect to “general fatigue” and the “fatigue” credit score in the EORTC QLQ‐C30. It is noteworthy that fatigue before the intervention was significantly lower in the participants performing HIIT than LMIE. Despite this significant lessening of chronic fatigue and exhaustion, the severity of symptoms was not reduced to the level of the general German population by multimodal rehabilitation with HIIT or LMIE (Schwarz and Hinz 2001). From this point of view, further studies on such therapeutic interventions for cancer‐related fatigue are warranted.

Although attractive as a time‐efficient alternative to more regular traditional exercise, it has been discussed that HIIT may discourage regular physical activity by untrained individuals (Hardcastle et al. 2014; del Vecchio et al. 2015). A sedentary population may either feel physically incapable not sufficiently motivated by avoidance of more exercise to participate in HIIT (Hardcastle et al. 2014). However, it was recently concluded that exercise of higher intensity helps sustain the motivation of cancer survivors to exercise (Martin et al. 2015). In the present context, both HIIT and LMIE increased reported quality of life without any signs of mental or physical overload. The fact that HIIT results in temporary exhaustion might actually improve the self‐confidence required to manage physically demanding activities of daily living.

Although our study population was rather small, a small number of participants allow to control potential side effects. We are well aware that a longer intervention might have produced a better health outcome. However, the duration chosen here is in accordance with the German insurance guidelines. Finally, the multimodal approach itself might have influenced the outcome. Since all patients in both groups were motivated to receive further education regarding nutrition, psychology, and general health, as well as to engage in occupational therapy, social counseling, relaxation, and aftercare, we cannot be certain that the outcome was a direct consequence of the HIIT or LMIE.

Finally, short‐term responses to 3 weeks of HIIT and LMIE were examined here and the results cannot necessarily be extrapolated to long‐term responses.

## Conclusion

The present controlled, randomized study demonstrates that a multimodal intervention including HIIT can be performed by female cancer survivors without adverse health effects. Here, HIIT and LMIE improved work economy, quality of life, and cancer‐related fatigue, but did not alter body composition or energy expenditure. Since the outcomes were similar with HIIT and LMIE, but the former takes less time, HIIT may be a time‐efficient strategy for improving certain psychophysiological aspects of health. However, studies involving more session of HIIT are required to determine its long‐term effects.

## Conflict of Interest

None declared.
